# Crystal structure of 2α-(1,1-di­phenyl­eth­yl)-4-methyl-4α,5α-diphenyl-1,3-dioxolane: the result of a non-acid pinacol rearrangement

**DOI:** 10.1107/S2056989015017752

**Published:** 2015-10-03

**Authors:** Richard M. Kirchner, Peter W. R. Corfield, Michelle Annabi, John Regan, Kevin Speina, Anthony DiProperzio, James A. Ciaccio, Joseph F. Capitani

**Affiliations:** a Department of Chemistry and Biochemistry, Manhattan College, 4513 Manhattan College Pkwy, Bronx NY 10471, USA; b Department of Chemistry, Fordham University, 441 East Fordham Road, Bronx, NY 10458, USA

**Keywords:** crystal structure, 1,3-dioxolane, non-acid-catalyzed pinacol rearrangement, acetal, C—H⋯O hydrogen bonds, C—H⋯π inter­actions, density functional analysis.

## Abstract

The title compound, C_30_H_28_O_2_, was obtained during recrystallization of (±)-1,2-diphenyl-1,2-propane­diol in 1-butanol, from an unexpected non-acid-catalyzed pinacol rearrangement followed by acetal formation of the newly formed aldehyde with the diol. The tri-substituted dioxolane ring has a twist conformation on the C—O bond opposite the methyl-substituted C atom. There is an intra­molecular C—H⋯π inter­action present involving one of the di­phenyl­ethyl rings and an H atom of the phenyl ring in position 4 of the dioxolane ring. In the crystal, mol­ecules are linked by weak C—H⋯O hydrogen bonds, forming chains along [001]. The chains are linked by a second C—H⋯π inter­action, forming sheets parallel to the *bc* plane.

## Abstract 2

The title compound was produced unexpectedly from (±)-1,2-diphenyl-1,2-propane­diol by a sequential non-acid Pinacol rearrangement followed by acetal formation during recrystallization in 1-butanol. The tri-substituted dioxolane ring has a twist conformation and in the crystal, mol­ecules are linked by C—H⋯O hydrogen bonds, forming chains along [001].

## Chemical context   

The pinacol rearrangement is a well-documented reaction (Collins, 1960 ▸) that converts substituted 1,2-diols into aldehydes or ketones (pinacolone derivatives), usually with the aid of mineral or Lewis acid catalysis. In the present work, a pinacol rearrangement has occurred during recrystallization **in the absence of a catalyst**, thus transforming the intended object of our study (**1**), into the pinacol rearrangement aldehyde (**3**), which then reacts (by acetal formation) with another mol­ecule of (**1**) to form the unexpected product (**2**) presented in this paper, as shown in the scheme below. 
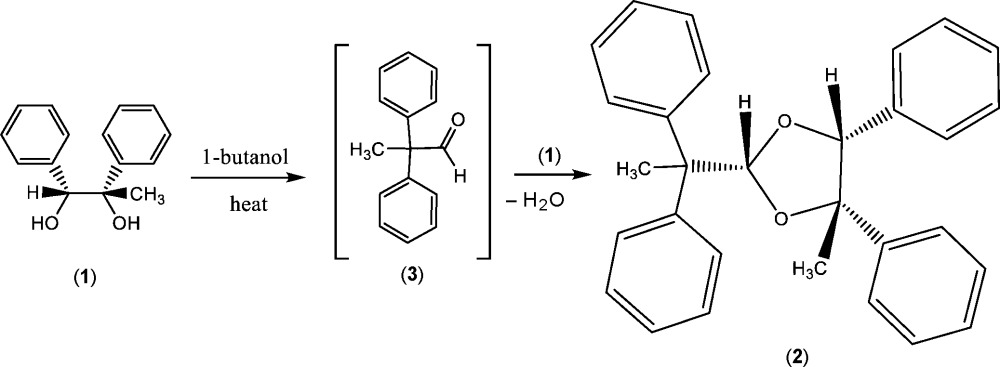


The pseudo-equatorial orientation of the di­phenyl­ethane group at C2 likely follows from thermodynamic control during the acetalization step. For the reaction conditions of our recrystallization, the acetalization step must proceed faster than pinacol rearrangement. A similar reaction has been described by Ciminale *et al.* (2005 ▸). There are very few other reports of the pinacol rearrangement occurring in the absence of catalysts: for example, the thermal rearrangement of pinacol to pinacolone in supercritical H_2_O (Ikushima *et al.*, 2000 ▸), the conversion of 1,1,2-tri­phenyl­ethane-1,2-diol to 1,2,2-tri­phenyl­ethan-1-one when heated above its melting point (Collins, 1960 ▸), and a vinyl­ogous pinacol rearrangement thermally induced in the solid state (Sekiya *et al.*, 2000 ▸).

## Structural commentary   

**Figure 1:**
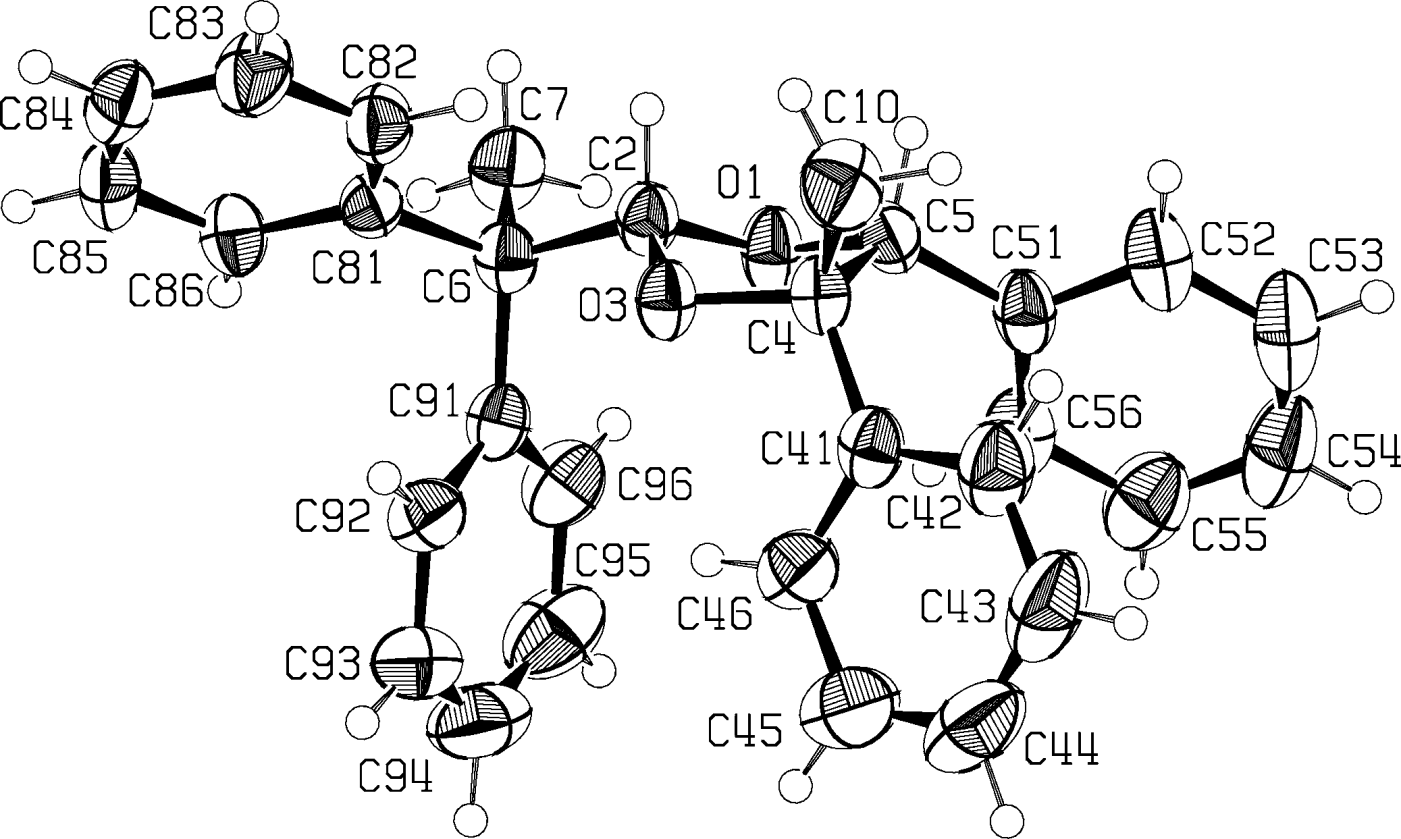
The mol­ecular structure of the title compound, (**2**), with atom labeling. Displacement ellipsoids are drawn at the 50% probability level. One of the H atoms on the methyl group C10 was omitted for clarity.

The mol­ecular structure of the title compound, (**2**), is illus­trated in Fig. 1 ▸. The dioxolane five-membered ring has a twist configuration on bond O1—C2, with atoms O1 and C2 at distances of 0.314 (4) and −0.330 (3) Å above and below the plane through atoms O3/C4/C5. The dioxolane ring has bond angles and distances that are within *ca* 3σ (using the larger s.u. values from the reported structures) of the values found in published X-ray structures (see for example: Rao & Hong Chan, 2014 ▸; Jones *et al.*, 1998 ▸). The planes of the two phenyl substituents on the dioxolane ring are inclined to one another by 44.67 (13)°. They and the di­phenyl­ethyl substituent are all *cis* to one another, in equatorial positions. The phenyl rings of the di­phenyl­ethyl substituent are inclined to one another by 68.16 (12)°. There is an intra­molecular C—H⋯π inter­action present involving one of the di­phenyl­ethyl rings (C91–C96) and an H atom of the phenyl ring in position 4 of the dioxolane ring (Table 1 ▸).

## Supra­molecular features   

**Figure 2:**
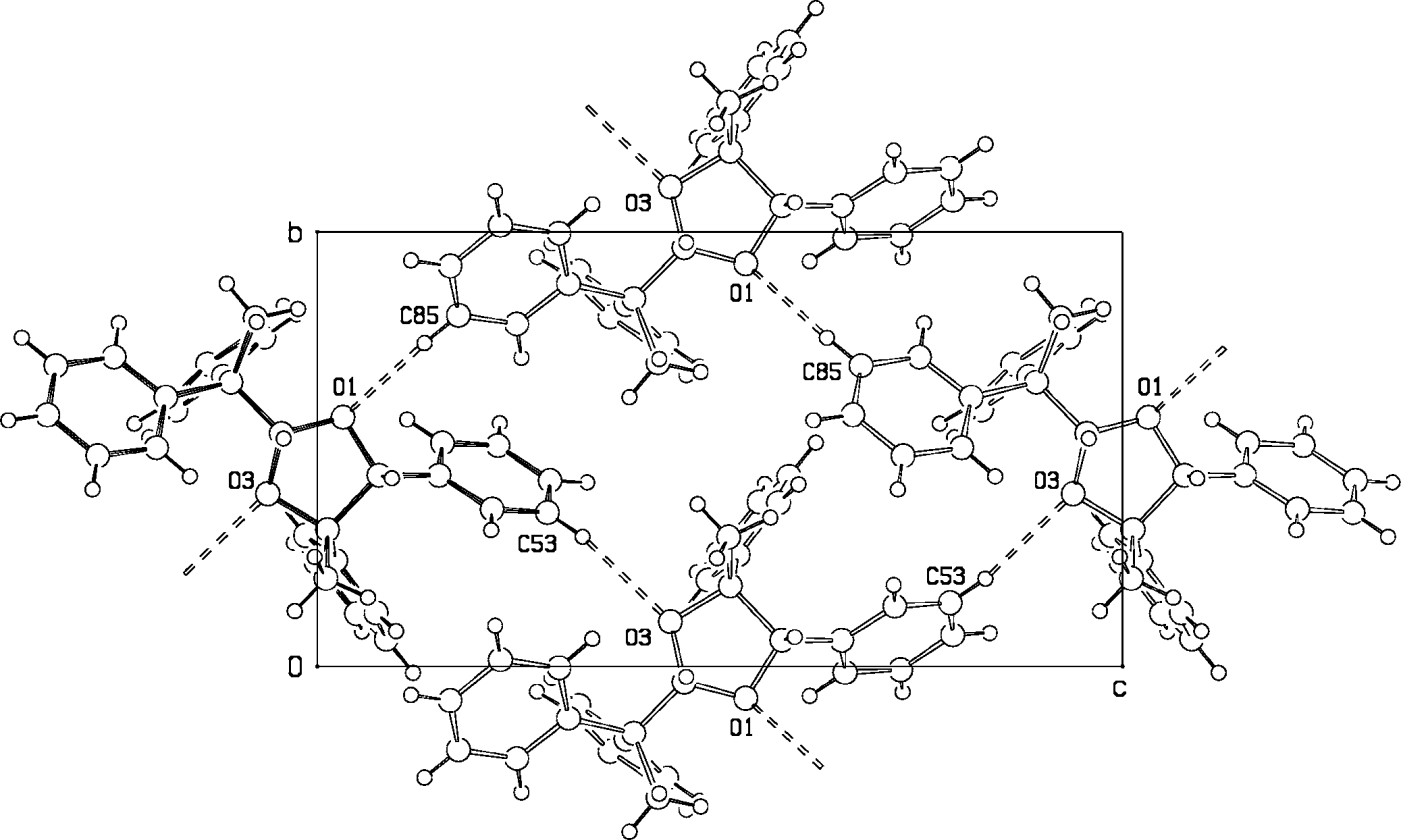
A view in projection along the *a* axis of the crystal packing of the title compound, (**2**). The C—H⋯O hydrogen bonds are shown as double dashed lines.

In the crystal, mol­ecules are linked by weak C—H⋯O hydrogen bonds, forming chains along [001]. The chains are linked by C—H⋯O bonds and by type I C—H⋯π inter­actions (Malone *et al.*, 1997 ▸), forming sheets parallel to the *bc* plane (Table 1 ▸ and Fig. 2 ▸).

## Database survey   

The Cambridge Structural Database (Version 5.36, last update February 2015; Groom & Allen 2014 ▸) was searched for structures containing the dioxolane ring. As there are several thousand dioxolane entries in the database, we selected only entries with the ring atoms plus one H atom each on C2 and C5, which includes the present structure. This search generated 594 hits, with 2227 sets of ring conformational angles that were reduced to 770 after removal of duplicates. There were 28 structures, 4% of the total, that contained near planar dioxolane rings, defined as rings where all torsional angles were less than 16°. Five-membered dioxolane rings have been described as ‘puckered envelopes’, ‘half-chair’ or ‘twisted’. Arbitrarily broad criteria were used for envelope or twist conformations. Structures were identified as **envelope** when one torsional angle was less than 10° and at least 10° less than the remaining angles, or **twist** when two torsional angles were below 20°, with less than 10° difference between them. In this way, all of the remaining structures could be classified as envelope (447 structures, or 58%) or twist (295 structures, or 38%). The envelope flap was most often one of the ring oxygen atoms (309 structures). In the twist structures, the twisted bond was usually either of the O1—C2 type (145 examples), as in the structure described in this paper, or of the O1–C5 type (123 examples). Of the twist structures, there were 39 like the present structure (**2**), close to an ideal symmetric twist configuration, where the two smallest torsional angles are within 3° of each other.

The wide variety of dioxolane ring conformations found in the structural literature reflects well the conclusion from our own calculations (see: Sections 5 and 6 below) as well as in Willy *et al.* (1970 ▸), that the dioxolane ring is highly flexible.

## Density functional analysis   

A B3LYP/6-311+G(d,p) density functional calculation (Spartan, 2006 ▸) of the present mol­ecule in the gas phase shows minimum energy for a twist configuration with similar torsional angles to those in the structure presented here. A calculation where H atoms replace phenyl and di­phenyl­ethyl substituents on the dioxolane ring suggests that the large phenyl rings have little effect upon the ring conformation (Table 2 ▸).

## Conformational analysis of 1,3-dioxolane rings   

**Figure 3:**
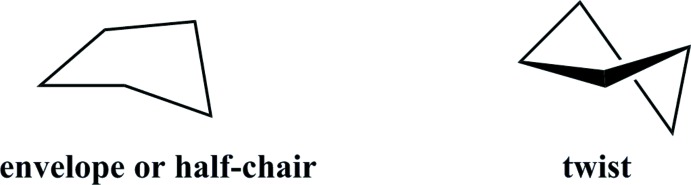
Ideal five-membered ring conformations.

No organic five-membered ring is exactly planar because flat rings would have eclipsed C—C bonds that can have considerable torsional strain. Five-membered rings are usually identified as envelope or half-chair with more or less distortion. The planar part of the ring is described by a least-squares fit of three or four atoms in the ring, or by the torsional angle formed by four contingent atoms in the ring. When only one of the remaining atoms is a significant distance from the plane, this conformation is described as an ‘envelope’. The non-planar atom defines the flap of the envelope. The torsional angles of an ideal half-chair configuration have two small angles of a given sign, two medium angles of opposite sign, and a single large angle of the same sign as the first. The Database Survey reveals that any atom in the ring can be the flap atom. When two atoms (one up, the other down) are a significant but different distance from the plane through the other three atoms, the conformation can be described as ‘distorted envelope’. When two atoms have equal significant distances in opposite directions from the plane, the ring conformation can be described as ‘twist’, as shown in Fig. 3 ▸. A full range of conformations from ideal envelope to ideal twist is obtained with various substituents on the ring because the various ring conformations do not differ substanti­ally in conformational energy (Willy *et al.* 1970 ▸).

**Figure 4:**
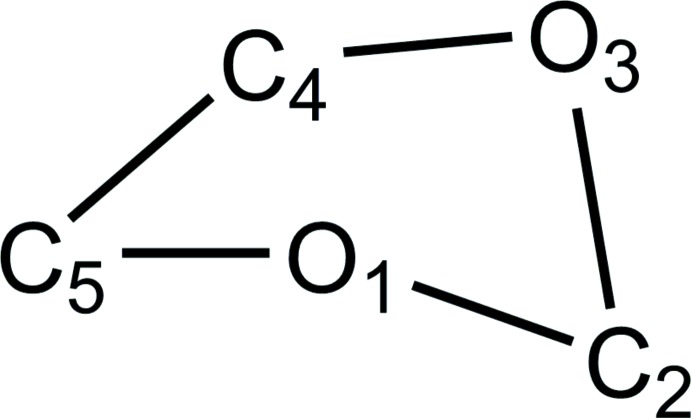
Atom numbering in the 1,3-dioxolane ring.

**Figure 5:**
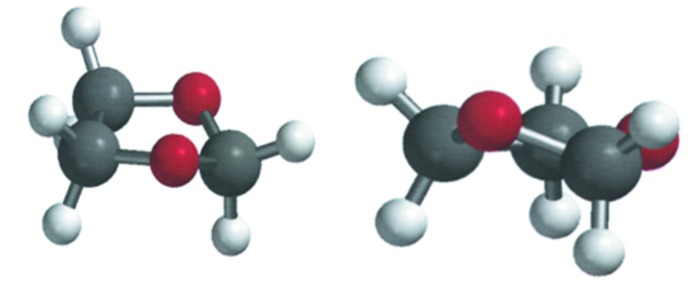
Perspective views of the dioxolane ring with hydrogen atoms as calculated with *Spartan*. Left: viewed as a distorted envelope. Right: viewed as twist.

**Figure 6:**
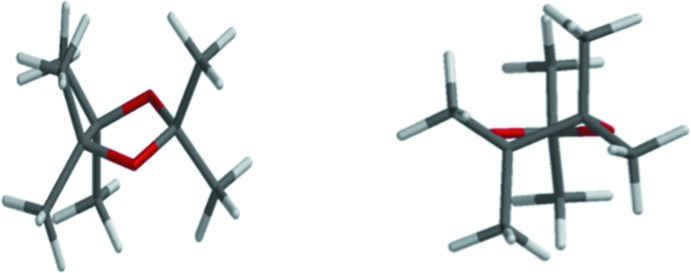
Perspective views of dioxolane ring with methyl groups as calculated with *Spartan*. Left: viewed as distorted envelope. Right: viewed as twist.

**Figure 7:**
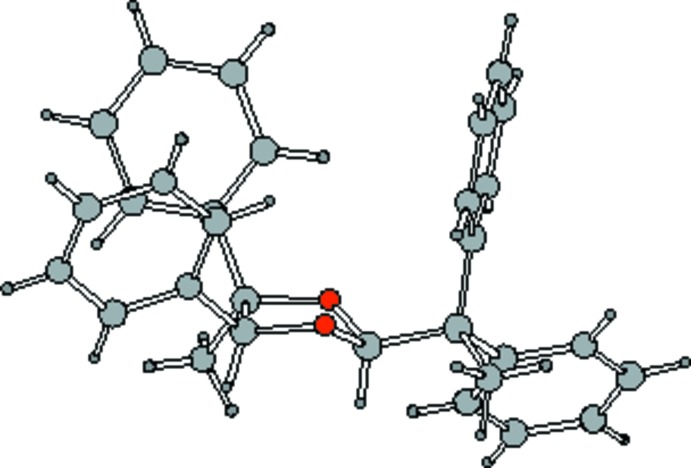
Perspective view of the X-ray structure of the title compound, (**2**).

To illustrate the conformational properties of the five-membered 1,3-dioxolane ring of the title compound, some B3LYP/6-311+G(d,p) density functional calculation results (Spartan, 2006 ▸) are given in Table 2 ▸. The atom numbering is shown in Fig. 4 ▸. Column two of the table (Ring with H atoms) shows a pattern of dihedral angles similar to the near-perfect twist found in the present crystal structure, shown in column five, where O1—C2 is the twisted bond. Fig. 5 ▸ above offers two views of the density functional theory (DFT) optimized structure. The pattern shown in column three (Ring with CH_3_ groups), has a much larger (O3—C4—C5—O1) torsional angle. The calculated conformation is still that of a twist, but the twist bond in this CH_3_ model is C4—C5, not O1—C2. The best plane through O1—C2—O3 has C4 + 0.28 Å above the plane and C5 − 0.28 Å below the plane, giving the CH_3_ model an ideal twist conformation. The DFT-optimized CH_3_-substituted structure is depicted in Fig. 6 ▸. Column four of Table 2 ▸ shows the DFT results for the title compound. The predicted conformation is similar to the conformation found in the crystal structure. The differences between columns four and five are presumably due to packing (inter­molecular) forces present in the X-ray structure. The Spartan DFT calculations do not include inter­molecular forces, but are calculations in the gas phase. Comparing distance and angle values in column two (dioxolane ring with only H atom substituents) to columns four and five (dioxolane rings with phenyl and di­phenyl­ethyl substituents) suggests these larger substituents have little effect upon the ring conformation. Fig. 7 ▸ views the title compound as a distorted envelope.

## Synthesis and crystallization   

A sample of (±)-1,2-diphenyl-1,2-propane­diol (Ciaccio *et al.*, 2001 ▸) was recrystallized in 1-butanol, as well as 2-butanol and 1-octa­nol. The solutions were mildly heated to obtain saturated solutions, cooled to room temperature and layered over water in an open test tube. In attempts to better characterize the rearrangements that occurred, we also recrystallized the starting material at the reflux temperature of 1-butanol. Thin layer chromatography revealed that the non-acid-catalyzed pinacol rearrangement was substanti­ally complete after 8 h, and that other unknown products were also present. The experimental density of a typical recrystallization product, determined by flotation, is 1.054 g ml^−1^. The melting point range was 435.9–443.2 K. Redoing the experimental density and melting point with hand-selected crystals with the same morphology as the X-ray data crystal gave values of 1.203 g ml^−1^ and 445.4–448.4 K, respectively.

The proton NMR spectrum was obtained with a Bruker AVANCE-400 NMR spectrometer using hand-picked crystals having the same morphology as the crystal used for the X-ray study. ^1^H NMR (400 MHz, CDCl_3_): δ 7.46 (*d*, 2H), 7.39 (*d*, 2H), 7.3–7.2 (*m*, 6H), 7.0-6.6 (*m*, 8H), 6.44 (*d*, 2H), 5.83 (*s*, 1H), 4.95 (*s*, 1H), 1.97 (*s*, 3H), 1.83 (*s*, 3H).

## Refinement details   

Crystal data, data collection and structure refinement details are summarized in Table 3 ▸. The H atoms were included in calculated positions and treated as riding atoms: C—H = 0.96-0.98 Å with U_iso_(H) = 1.5U_eq_(C) for methyl H atoms, U_eq_(C) for methine H atoms, and 1.2U_eq_(C) for other H atoms.

## Supplementary Material

Crystal structure: contains datablock(s) I. DOI: 10.1107/S2056989015017752/su5202sup1.cif

Structure factors: contains datablock(s) I. DOI: 10.1107/S2056989015017752/su5202Isup2.hkl

Supporting information file. DOI: 10.1107/S2056989015017752/su5202Isup3.cml

CCDC reference: 1426279

Additional supporting information:  crystallographic information; 3D view; checkCIF report

## Figures and Tables

**Table 1:** Hydrogen-bond geometry (, ) *Cg*3 and *Cg*5 are the centroids of the C51C56 and C91C96 rings, respectively.

*D*H*A*	*D*H	H*A*	*D* *A*	*D*H*A*
C53H53O3^i^	0.93	2.61	3.533(3)	170
C85H85O1^ii^	0.93	2.50	3.411(3)	167
C46H46*Cg*5	0.93	2.99	3.894(3)	164
C86H86*Cg*3^ii^	0.93	2.91	3.799(2)	160

Symmetry codes: (i) 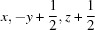; (ii) 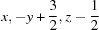.

**Table 2:** Substituted 1,3-dioxolanes (, ) Dioxolane is the title compound (**2**). The phenyl and diphenyl substituents are replaced by H atoms in column two, and CH_3_ groups in column three.

Parameter	Ring with H atoms	Ring with CH_3_ groups	Dioxolane	X-ray Dioxolane
Bond length				
O1C2	1.41	1.43	1.39	1.406(2)
C2O3	1.41	1.43	1.39	1.408(2)
O3C4	1.43	1.45	1.42	1.444(2)
C4C5	1.55	1.57	1.59	1.577(2)
C5O1	1.43	1.45	1.40	1.427(2)
				
Bond angle				
O1C2O3	106.3	105.7	104.4	104.5(1)
C2O3O4	106.3	110.0	108.6	106.9(1)
O3C4C5	104.3	101.3	101.5	102.2(1)
C4C5O1	103.8	101.3	102.9	103.0(1)
C5O1C2	104.5	110.0	105.8	103.4(1)
				
Torsion angle				
O1C2O3C4	33.1	11.8	35.6	37.59(2)
C2O3C4C5	13.3	28.1	15.0	14.28(2)
O3C4C5O1	10.3	33.2	10.1	13.50(1)
C4C5O1C2	29.9	28.1	31.6	35.50(1)
C5O1C2O3	39.9	11.8	42.4	46.21(2)
				
Distance from plane				
C2O3/C4/C5	0.31	0.64	+0.34	0.330(3)
O1O3/C4/C5	+0.25	0.78	0.24	+0.314(4)
C4O1/C2/O3		+0.28		
C5O1/C2/O3		0.28		

**Table 3:** Experimental details

Crystal data	
Chemical formula	C_30_H_28_O_2_
*M*_r_	420.52
Crystal system, space group	Monoclinic, *P*2_1_/*c*
Temperature (K)	302
*a*, *b*, *c* ()	16.720(3), 9.0056(9), 16.6747(12)
()	112.040(9)
*V* (^3^)	2327.3(5)
*Z*	4
Radiation type	Mo *K*
(mm^1^)	0.07
Crystal size (mm)	0.4 0.4 0.26

Data collection	
Diffractometer	EnrafNonius CAD-4
No. of measured, independent and observed [*I* > 2(*I*)] reflections	6377, 4547, 2612
*R*_int_	0.025
(sin /)_max_ (^1^)	0.616

Refinement	
*R*[*F* ^2^ > 2(*F* ^2^)], *wR*(*F* ^2^), *S*	0.047, 0.133, 1.03
No. of reflections	4547
No. of parameters	291
H-atom treatment	H-atom parameters constrained
_max_, _min_ (e ^3^)	0.16, 0.18

Computer programs: *CAD-4 EXPRESS* (EnrafNonius, 1994 ▸), *SUPERFLIP* (Palatinus Chapuis, 2007 ▸), *SHELXL97* (Sheldrick, 2008 ▸) and *ORTEPIII* (Burnett Johnson, 1996 ▸). Data reduction followed procedures in Corfield *et al.* (1973 ▸) and data were averaged with a local version of *SORTAV* (Blessing, 1989 ▸),

## supplementary crystallographic information

### Crystal data

**Table:**

C_30_H_28_O_2_	*F*(000) = 896
*M**_r_* = 420.52	*D*_x_ = 1.200 Mg m^−^^3^
Monoclinic, *P*2_1_/*c*	Melting point = 436–443 K
Hall symbol: -P 2ybc	Mo *K*α radiation, λ = 0.71073 Å
*a* = 16.720 (3) Å	Cell parameters from 25 reflections
*b* = 9.0056 (9) Å	θ = 4.5–10.1°
*c* = 16.6747 (12) Å	µ = 0.07 mm^−^^1^
β = 112.040 (9)°	*T* = 302 K
*V* = 2327.3 (5) Å^3^	Block, colourless
*Z* = 4	0.4 × 0.4 × 0.26 mm

### Data collection

**Table:**

Enraf–Nonius CAD-4 diffractometer	*R*_int_ = 0.025
Radiation source: fine-focus sealed tube	θ_max_ = 26.0°, θ_min_ = 2.5°
Graphite monochromator	*h* = −20→19
θ/2θ scans	*k* = 0→11
6377 measured reflections	*l* = 0→20
4547 independent reflections	3 standard reflections every 180 min
2612 reflections with *I* > 2σ(*I*)	intensity decay: 3.1(8)

### Refinement

**Table:**

Refinement on *F*^2^	Primary atom site location: structure-invariant direct methods
Least-squares matrix: full	Secondary atom site location: difference Fourier map
*R*[*F*^2^ > 2σ(*F*^2^)] = 0.047	Hydrogen site location: inferred from neighbouring sites
*wR*(*F*^2^) = 0.133	H-atom parameters constrained
*S* = 1.03	*w* = 1/[σ^2^(*F*_o_^2^) + (0.020*P*)^2^ + 0.5*P*] where *P* = (*F*_o_^2^ + 2*F*_c_^2^)/3
4547 reflections	(Δ/σ)_max_ < 0.001
291 parameters	Δρ_max_ = 0.16 e Å^−^^3^
0 restraints	Δρ_min_ = −0.18 e Å^−^^3^

### Special details

**Table:**

Geometry. All e.s.d.'s (except the e.s.d. in the dihedral angle between two l.s. planes) are estimated using the full covariance matrix. The cell e.s.d.'s are taken into account individually in the estimation of e.s.d.'s in distances, angles and torsion angles; correlations between e.s.d.'s in cell parameters are only used when they are defined by crystal symmetry. An approximate (isotropic) treatment of cell e.s.d.'s is used for estimating e.s.d.'s involving l.s. planes.
Refinement. In the monoclinic unit cell, the *a* and *c* axes are of very similar lengths, so that before data collection commenced, it was important to check that the Laue symmetry was indeed 2/m and not mmm. This was accomplished by temporarily transforming the cell to orthorhombic axes, and collecting all 8 forms of the (orthorhombic) 111 and 222 reflections. In each case, the 8 forms clearly split into two different sets of four, verifying the monoclinic symmetry.

### Fractional atomic coordinates and isotropic or equivalent isotropic displacement parameters (Å^2^)

**Table:**

	*x*	*y*	*z*	*U*_iso_*/*U*_eq_	
O1	0.18420 (9)	0.57319 (14)	0.10104 (8)	0.0484 (4)	
O3	0.19021 (8)	0.39714 (14)	0.00987 (8)	0.0419 (3)	
C2	0.15272 (14)	0.5361 (2)	0.01270 (12)	0.0416 (5)	
H2	0.0899	0.5249	−0.0083	0.042*	
C4	0.19172 (13)	0.3148 (2)	0.08470 (12)	0.0419 (5)	
C41	0.27953 (14)	0.2435 (2)	0.12749 (12)	0.0448 (5)	
C42	0.29066 (18)	0.1231 (3)	0.18187 (15)	0.0669 (7)	
H42	0.2430	0.0822	0.1897	0.080*	
C43	0.3712 (2)	0.0626 (3)	0.22479 (17)	0.0879 (9)	
H43	0.3775	−0.0186	0.2612	0.105*	
C44	0.4421 (2)	0.1213 (4)	0.21416 (18)	0.0874 (9)	
H44	0.4966	0.0810	0.2435	0.105*	
C45	0.43208 (17)	0.2395 (3)	0.16012 (17)	0.0770 (8)	
H45	0.4799	0.2790	0.1520	0.092*	
C46	0.35148 (15)	0.3013 (3)	0.11716 (14)	0.0586 (6)	
H46	0.3458	0.3826	0.0810	0.070*	
C5	0.17302 (14)	0.4396 (2)	0.14164 (12)	0.0441 (5)	
H5	0.1123	0.4325	0.1348	0.044*	
C51	0.22718 (15)	0.4399 (2)	0.23635 (12)	0.0459 (5)	
C52	0.19966 (18)	0.3615 (3)	0.29192 (14)	0.0636 (7)	
H52	0.1466	0.3134	0.2707	0.076*	
C53	0.2504 (2)	0.3536 (3)	0.37949 (16)	0.0801 (8)	
H53	0.2321	0.2978	0.4164	0.096*	
C54	0.3268 (2)	0.4272 (3)	0.41131 (16)	0.0814 (9)	
H54	0.3605	0.4228	0.4701	0.098*	
C55	0.35420 (18)	0.5080 (3)	0.35694 (16)	0.0758 (8)	
H55	0.4063	0.5591	0.3790	0.091*	
C56	0.30502 (16)	0.5139 (3)	0.26954 (14)	0.0588 (6)	
H56	0.3244	0.5680	0.2329	0.071*	
C6	0.17518 (13)	0.6541 (2)	−0.04152 (11)	0.0386 (5)	
C7	0.13530 (15)	0.8014 (2)	−0.02620 (14)	0.0543 (6)	
H7A	0.1514	0.8808	−0.0555	0.081*	
H7B	0.1562	0.8222	0.0347	0.081*	
H7C	0.0736	0.7926	−0.0483	0.081*	
C81	0.13165 (12)	0.6192 (2)	−0.13860 (11)	0.0373 (5)	
C82	0.07434 (14)	0.5033 (2)	−0.17192 (13)	0.0514 (6)	
H82	0.0636	0.4371	−0.1344	0.062*	
C83	0.03285 (15)	0.4846 (3)	−0.26028 (14)	0.0610 (6)	
H83	−0.0060	0.4069	−0.2814	0.073*	
C84	0.04836 (15)	0.5791 (3)	−0.31673 (14)	0.0560 (6)	
H84	0.0199	0.5667	−0.3761	0.067*	
C85	0.10630 (15)	0.6922 (2)	−0.28491 (13)	0.0554 (6)	
H85	0.1182	0.7559	−0.3229	0.066*	
C86	0.14699 (14)	0.7122 (2)	−0.19714 (12)	0.0478 (5)	
H86	0.1858	0.7903	−0.1767	0.057*	
C91	0.27312 (13)	0.6670 (2)	−0.01420 (12)	0.0417 (5)	
C92	0.31673 (14)	0.5900 (2)	−0.05657 (14)	0.0518 (6)	
H92	0.2862	0.5266	−0.1017	0.062*	
C93	0.40453 (15)	0.6047 (3)	−0.03367 (16)	0.0679 (7)	
H93	0.4322	0.5522	−0.0638	0.082*	
C94	0.45124 (17)	0.6955 (3)	0.03284 (19)	0.0790 (8)	
H94	0.5104	0.7064	0.0476	0.095*	
C95	0.41022 (19)	0.7700 (3)	0.07731 (18)	0.0783 (8)	
H95	0.4419	0.8300	0.1237	0.094*	
C96	0.32217 (16)	0.7572 (3)	0.05420 (14)	0.0599 (6)	
H96	0.2952	0.8099	0.0849	0.072*	
C10	0.11855 (16)	0.2027 (3)	0.05553 (15)	0.0652 (7)	
H10A	0.0660	0.2516	0.0205	0.098*	
H10B	0.1118	0.1596	0.1053	0.098*	
H10C	0.1316	0.1259	0.0223	0.098*	

### Atomic displacement parameters (Å^2^)

**Table:**

	*U*^11^	*U*^22^	*U*^33^	*U*^12^	*U*^13^	*U*^23^
O1	0.0752 (10)	0.0384 (8)	0.0376 (8)	0.0104 (7)	0.0280 (7)	0.0030 (6)
O3	0.0587 (9)	0.0362 (7)	0.0341 (7)	0.0042 (7)	0.0212 (6)	0.0009 (6)
C2	0.0501 (12)	0.0423 (11)	0.0353 (11)	0.0070 (10)	0.0192 (9)	0.0005 (9)
C4	0.0543 (13)	0.0391 (11)	0.0353 (10)	−0.0009 (10)	0.0201 (9)	0.0035 (9)
C41	0.0597 (14)	0.0408 (11)	0.0351 (10)	0.0052 (11)	0.0192 (10)	−0.0053 (9)
C42	0.0881 (19)	0.0587 (15)	0.0575 (14)	0.0193 (14)	0.0313 (14)	0.0118 (13)
C43	0.116 (3)	0.081 (2)	0.0603 (17)	0.043 (2)	0.0258 (18)	0.0202 (15)
C44	0.080 (2)	0.099 (2)	0.0642 (17)	0.041 (2)	0.0058 (16)	−0.0043 (17)
C45	0.0579 (17)	0.093 (2)	0.0720 (17)	0.0099 (16)	0.0146 (14)	−0.0103 (17)
C46	0.0570 (16)	0.0611 (15)	0.0532 (13)	0.0058 (13)	0.0156 (12)	−0.0017 (12)
C5	0.0502 (12)	0.0476 (12)	0.0418 (11)	0.0040 (10)	0.0256 (10)	0.0055 (10)
C51	0.0666 (15)	0.0406 (11)	0.0386 (11)	0.0077 (11)	0.0289 (11)	0.0003 (10)
C52	0.0938 (19)	0.0587 (15)	0.0499 (14)	−0.0016 (14)	0.0401 (13)	0.0036 (12)
C53	0.133 (3)	0.0688 (17)	0.0516 (15)	0.0109 (19)	0.0500 (17)	0.0131 (14)
C54	0.118 (3)	0.0799 (19)	0.0395 (14)	0.0189 (19)	0.0215 (16)	0.0017 (14)
C55	0.0838 (19)	0.0815 (19)	0.0527 (16)	0.0003 (16)	0.0148 (14)	−0.0081 (14)
C56	0.0742 (17)	0.0582 (14)	0.0469 (13)	0.0010 (13)	0.0261 (12)	0.0010 (11)
C6	0.0482 (12)	0.0341 (10)	0.0358 (10)	0.0042 (9)	0.0185 (9)	0.0009 (8)
C7	0.0690 (15)	0.0445 (12)	0.0512 (13)	0.0141 (11)	0.0246 (11)	0.0017 (10)
C81	0.0391 (11)	0.0370 (10)	0.0376 (10)	0.0061 (9)	0.0166 (9)	0.0044 (9)
C82	0.0552 (13)	0.0549 (13)	0.0423 (12)	−0.0080 (12)	0.0163 (10)	0.0081 (11)
C83	0.0616 (15)	0.0645 (15)	0.0467 (13)	−0.0164 (12)	0.0086 (11)	−0.0014 (12)
C84	0.0629 (15)	0.0630 (15)	0.0364 (11)	0.0050 (13)	0.0122 (11)	0.0030 (11)
C85	0.0750 (16)	0.0526 (14)	0.0432 (12)	0.0056 (13)	0.0275 (12)	0.0123 (11)
C86	0.0585 (14)	0.0432 (12)	0.0450 (12)	−0.0043 (11)	0.0231 (10)	0.0027 (10)
C91	0.0507 (13)	0.0354 (10)	0.0372 (10)	0.0011 (10)	0.0143 (9)	0.0040 (9)
C92	0.0471 (14)	0.0550 (13)	0.0491 (12)	0.0028 (11)	0.0131 (10)	−0.0005 (11)
C93	0.0479 (15)	0.0834 (18)	0.0709 (16)	0.0079 (14)	0.0205 (13)	0.0019 (15)
C94	0.0451 (15)	0.084 (2)	0.093 (2)	−0.0030 (15)	0.0084 (15)	0.0103 (17)
C95	0.070 (2)	0.0670 (17)	0.0729 (17)	−0.0140 (15)	−0.0023 (15)	−0.0068 (15)
C96	0.0648 (16)	0.0535 (14)	0.0530 (14)	−0.0017 (12)	0.0125 (12)	−0.0055 (11)
C10	0.0717 (17)	0.0556 (14)	0.0643 (15)	−0.0153 (13)	0.0210 (13)	0.0030 (12)

### Geometric parameters (Å, º)

**Table:**

O1—C2	1.406 (2)	C56—H56	0.9300
O1—C5	1.427 (2)	C6—C91	1.531 (3)
O3—C2	1.408 (2)	C6—C81	1.538 (3)
O3—C4	1.444 (2)	C6—C7	1.549 (3)
C2—C6	1.531 (3)	C7—H7A	0.9600
C2—H2	0.9800	C7—H7B	0.9600
C4—C41	1.513 (3)	C7—H7C	0.9600
C4—C10	1.519 (3)	C81—C86	1.381 (3)
C4—C5	1.577 (3)	C81—C82	1.385 (3)
C41—C42	1.381 (3)	C82—C83	1.383 (3)
C41—C46	1.379 (3)	C82—H82	0.9300
C42—C43	1.379 (4)	C83—C84	1.364 (3)
C42—H42	0.9300	C83—H83	0.9300
C43—C44	1.369 (4)	C84—C85	1.368 (3)
C43—H43	0.9300	C84—H84	0.9300
C44—C45	1.364 (4)	C85—C86	1.375 (3)
C44—H44	0.9300	C85—H85	0.9300
C45—C46	1.384 (3)	C86—H86	0.9300
C45—H45	0.9300	C91—C92	1.378 (3)
C46—H46	0.9300	C91—C96	1.390 (3)
C5—C51	1.497 (3)	C92—C93	1.378 (3)
C5—H5	0.9800	C92—H92	0.9300
C51—C52	1.374 (3)	C93—C94	1.363 (4)
C51—C56	1.380 (3)	C93—H93	0.9300
C52—C53	1.388 (3)	C94—C95	1.361 (4)
C52—H52	0.9300	C94—H94	0.9300
C53—C54	1.358 (4)	C95—C96	1.379 (3)
C53—H53	0.9300	C95—H95	0.9300
C54—C55	1.368 (4)	C96—H96	0.9300
C54—H54	0.9300	C10—H10A	0.9600
C55—C56	1.380 (3)	C10—H10B	0.9600
C55—H55	0.9300	C10—H10C	0.9600
			
C2—O1—C5	103.44 (14)	C51—C56—H56	119.9
C2—O3—C4	106.93 (13)	C2—C6—C91	110.44 (15)
O1—C2—O3	104.48 (14)	C2—C6—C81	110.85 (15)
O1—C2—C6	112.05 (16)	C91—C6—C81	111.09 (15)
O3—C2—C6	112.72 (15)	C2—C6—C7	106.33 (15)
O1—C2—H2	109.2	C91—C6—C7	111.39 (16)
O3—C2—H2	109.2	C81—C6—C7	106.59 (15)
C6—C2—H2	109.2	C6—C7—H7A	109.5
O3—C4—C41	108.94 (15)	C6—C7—H7B	109.5
O3—C4—C10	108.30 (16)	H7A—C7—H7B	109.5
C41—C4—C10	113.07 (17)	C6—C7—H7C	109.5
O3—C4—C5	102.18 (14)	H7A—C7—H7C	109.5
C41—C4—C5	113.25 (16)	H7B—C7—H7C	109.5
C10—C4—C5	110.42 (17)	C86—C81—C82	117.24 (18)
C42—C41—C46	118.0 (2)	C86—C81—C6	118.70 (18)
C42—C41—C4	120.7 (2)	C82—C81—C6	124.00 (17)
C46—C41—C4	121.23 (19)	C83—C82—C81	120.9 (2)
C43—C42—C41	121.1 (3)	C83—C82—H82	119.5
C43—C42—H42	119.5	C81—C82—H82	119.5
C41—C42—H42	119.5	C84—C83—C82	120.7 (2)
C44—C43—C42	120.3 (3)	C84—C83—H83	119.7
C44—C43—H43	119.8	C82—C83—H83	119.7
C42—C43—H43	119.8	C83—C84—C85	119.1 (2)
C45—C44—C43	119.3 (3)	C83—C84—H84	120.4
C45—C44—H44	120.3	C85—C84—H84	120.4
C43—C44—H44	120.3	C84—C85—C86	120.4 (2)
C44—C45—C46	120.7 (3)	C84—C85—H85	119.8
C44—C45—H45	119.6	C86—C85—H85	119.8
C46—C45—H45	119.6	C85—C86—C81	121.6 (2)
C41—C46—C45	120.6 (2)	C85—C86—H86	119.2
C41—C46—H46	119.7	C81—C86—H86	119.2
C45—C46—H46	119.7	C92—C91—C96	116.9 (2)
O1—C5—C51	111.34 (17)	C92—C91—C6	121.47 (17)
O1—C5—C4	102.98 (14)	C96—C91—C6	121.64 (19)
C51—C5—C4	117.29 (17)	C91—C92—C93	121.6 (2)
O1—C5—H5	108.3	C91—C92—H92	119.2
C51—C5—H5	108.3	C93—C92—H92	119.2
C4—C5—H5	108.3	C94—C93—C92	120.6 (2)
C52—C51—C56	118.8 (2)	C94—C93—H93	119.7
C52—C51—C5	119.2 (2)	C92—C93—H93	119.7
C56—C51—C5	122.01 (18)	C95—C94—C93	119.1 (2)
C51—C52—C53	120.6 (3)	C95—C94—H94	120.4
C51—C52—H52	119.7	C93—C94—H94	120.4
C53—C52—H52	119.7	C94—C95—C96	120.7 (2)
C54—C53—C52	120.0 (2)	C94—C95—H95	119.7
C54—C53—H53	120.0	C96—C95—H95	119.7
C52—C53—H53	120.0	C95—C96—C91	121.1 (2)
C53—C54—C55	120.0 (2)	C95—C96—H96	119.4
C53—C54—H54	120.0	C91—C96—H96	119.4
C55—C54—H54	120.0	C4—C10—H10A	109.5
C54—C55—C56	120.4 (3)	C4—C10—H10B	109.5
C54—C55—H55	119.8	H10A—C10—H10B	109.5
C56—C55—H55	119.8	C4—C10—H10C	109.5
C55—C56—C51	120.2 (2)	H10A—C10—H10C	109.5
C55—C56—H56	119.9	H10B—C10—H10C	109.5
			
O1—C2—O3—C4	37.54 (19)	C4—C5—O1—C2	35.48 (18)
C2—O3—C4—C5	−14.20 (18)	C5—O1—C2—O3	−46.16 (18)
O3—C4—C5—O1	−13.06 (18)		

### Hydrogen-bond geometry (Å, º)

Cg3 and Cg5 are the centroids of the C51–C56 and C91–C96 rings, respectively.

**Table:**

*D*—H···*A*	*D*—H	H···*A*	*D*···*A*	*D*—H···*A*
C53—H53···O3^i^	0.93	2.61	3.533 (3)	170
C85—H85···O1^ii^	0.93	2.50	3.411 (3)	167
C46—H46···*Cg*5	0.93	2.99	3.894 (3)	164
C86—H86···*Cg*3^ii^	0.93	2.91	3.799 (2)	160

Symmetry codes: (i) *x*, −*y*+1/2, *z*+1/2; (ii) *x*, −*y*+3/2, *z*−1/2.
